# The Activation and Inactivation of Mature CD4 T cells: A Case for Peripheral Self–Nonself Discrimination

**DOI:** 10.1111/sji.12173

**Published:** 2014-05-21

**Authors:** P A Bretscher

**Affiliations:** University of SaskatchewanSaskatoon, Saskatchewan, Canada

## Abstract

The establishment of central tolerance to most self-antigens results in a repertoire of mature peripheral lymphocytes specific for foreign and peripheral self-antigens. The framework that single, mature lymphocytes are inactivated by antigen, whereas their activation requires lymphocyte cooperation, accounts for diverse observations and incorporates a mechanism of peripheral tolerance. This framework accounts for the generalizations that the sustained activation by antigen of most B cells and CD8 T cells requires CD4 T helper cells; in the absence of CD4 T cells, antigen can inactivate these B cells and CD8 T cells. In this sense, CD4 T cells are the guardians of the fate of most B cells and CD8 T cells when they encounter antigen. I argue here that the single-lymphocyte/multiple-lymphocyte framework for the inactivation/activation of lymphocytes also applies to CD4 T cells. I consider within this framework a model for the activation/inactivation of CD4 T cells that is consistent with the large majority of contemporary observations, including significant clinical observations. I outline the grounds why I feel this model is more plausible than the contemporary and predominant pathogen-associated molecular pattern (PAMP) and Danger Models for CD4 T cell activation. These models are based upon what I consider the radical premise that self–nonself discrimination does not exist at the level of mature CD4 T cells. I explain why I feel this feature renders the PAMP and Danger Models somewhat implausible. The model I propose, in contrast, is conservative in that it embodies such a process of self–nonself discrimination.

## Preface

The nature both of observations and of considerations in the contemporary immunological literature, invoked in trying to understand how the immune system functions, is different in kind from those prevalent 40 years ago. This is to be expected, as the tools at hand have radically changed. Nevertheless, the contemporary and predominant employment of observations and considerations at the molecular and cellular level, almost exclusive of those at the level of the system, results in the neglect of important observations and considerations at this level, some of them prominent in the older literature. I believe this neglect undermines our ability to understand how the immune system functions.

I focus in this and a related forum 1 on two fundamental questions concerning the immune system. This, the first forum, is directed at considering the question of how antigen interacts differently with mature lymphocytes to result in their activation and inactivation. This question bears on the contemporary issue of whether the immune system possesses the attribute of peripheral self–nonself discrimination at the level of CD4 T cells. The second question, addressed in the second forum, is, if activation occurs, what determines the subset of CD4 T cells generated, and so the class of immunity induced? I believe this second question is circumscribed by the alternative solutions we consider plausible when attempting to answer the first question, which is why I hope the reader can consider these forums together.

I understand the manuscript submitted was reviewed by three colleagues. Two of these, Colin Anderson (CA) and Alexandre Corthay (AC), responded by addressing some thoughtful questions to me, in some cases indicating disagreement. I wish to respond to these questions, to foster discussion, but I try not to be too elaborate, as this would render the few salient ideas I offer for consideration, less accessible. I have responded in two ways. The first is to modify the text, in an attempt to address relatively minor points that were made, to improve clarity. The second way deals with issues where the reviewer and I currently hold different perspectives, or the comments/questions were more extensive, and so a response is less straightforward. Rather subtle and involved considerations may apply, not to say a different knowledge of the enormous literature. We usually avoid a full description of our considerations leading to a view we hold. We try to make what we consider plausible attractive. I have decided to partially address serious considerations brought up by CA and AC by making either in various places comments put into (brackets), indicating both their concerns and a succinct response on my part, or in a section devoted to addressing their comments/questions.

## A context for the discussion of peripheral tolerance at the level of CD4 T cells

Erhlich first envisaged, in the early 1900s, that immunity against self-antigens would present, if it occurred, a grave threat to the individual 2. Observations, beginning in the late 1940s, led to the recognition of autoimmunity as a pathological state 3,4. Ideas on how autoimmunity might be prevented were a driving force in the formulation of the Clonal Selection Theory in the very late 1940s and throughout the 1950s. Burnet and Fenner were the first to propose that the immune system relied upon the early presence of self-antigens in ontogeny, that is, in the ‘history’ of the individual, to achieve unresponsiveness against these antigens 5. It is convenient to refer to this proposal as the ‘historical postulate’ 6. Jerne 7 and Talmage 8, as well as Burnet 9, adopted the historical postulate in their critical contributions to the development of the Clonal Selection Theory. Hashek 10 and Medawar and their colleagues 11 provided the first experimental evidence accepted as supporting Burnet and Fenner's vision. Lederberg gave in 1959 the clearest, most axiomatic and beautiful formulation of the Clonal Selection Theory 12. He envisaged that ‘precursor cells’ were generated throughout life. He therefore proposed that purging of precursor cells with anti-self-reactivity must also occur throughout life.

Lederberg's ideas set the scene for contemporary discussion. He envisaged that when precursor cells develop from stem cells, with surface receptors that can interact with antigen, they are ‘silenced’ on interacting with the antigen. These precursor cells, if they do not interact with antigen over a short time of a few days and so are not silenced, possess an *internal clock* that triggers their differentiation into cells, bearing the same receptors, but now reprogrammed so that when their receptors interact with antigen, they are activated, that is, they multiply and their progeny differentiate into antibody-producing or other effector cells. I refer to this model as ‘the internal clock’ model of lymphocyte inactivation/activation.

It became accepted, some decades after Lederberg's proposals were formulated, that they describe well the development of mature B cells 13 and mature T cells 14 with two related caveats. These two caveats are related to more modern findings. Self-antigens, present at sufficient levels in the primary lymphoid organs, where lymphocytes develop, purge the lymphocytes specific for these self-antigens by a Lederberg-type mechanism. This purging is referred to as ‘central tolerance’. Some self-antigens, typically and predominantly present in specialized organs, are not always present in primary lymphoid organs at the level required to reliably cause central tolerance. In the case of insulin, a target antigen in autoimmune diabetes, a low level of thymic expression, and so less thorough central deletion, corresponds to increased susceptibility to autoimmunity 15,16. Such antigens, not present in the thymus to reliably cause central tolerance, are referred to as ‘peripheral self-antigens’. The existence of peripheral self-antigens, in the context of various observations, led to the acceptance of the idea in the 1960s that mature lymphocytes could be either inactivated (silenced) or activated (induced) by antigen through competitive processes 17. This proposal that lymphocytes could be activated/inactivated through competitive processes was different from what Lederberg had envisaged in the late 1950s, as described previously. The idea that lymphocytes could be either activated or inactivated arose as a result of the findings in the 1960s that neonates were both competent to mount immune responses to certain antigens and could be rendered unresponsive to these same antigens by administering the antigen under different circumstances. Although the observations only indicated the existence of competitive processes, leading to antibody responses or ablating this possibility, it was generally envisaged that this competition took place at the level of the individual lymphocyte or antibody precursor cell 17.

Cohn and I proposed in 1970 that a single mature lymphocyte, on interacting with antigen, can be inactivated and that the activation of a lymphocyte requires the antigen to mediate interactions between this responding lymphocyte and other lymphocytes specific for the antigen 18. This model is conveniently referred to as the single-lymphocyte/multiple-lymphocyte model for the inactivation/activation of lymphocytes. We proposed that the interaction of the lymphocyte receptor with antigen generated a signal, signal 1, that, if generated alone for a sufficient length of time, inactivated the lymphocyte. Lymphocyte activation required the generation of signal 1 as well as the generation of signal 2. The generation of signal 2 followed the recognition of antigen by an interacting lymphocyte, usefully referred to as a helper lymphocyte. We proposed that the delivery of signal 2 involved a membrane/membrane interaction between the interacting cells and/or the release of short-range ‘lymphokines’ 18. I will refer to this as the ‘original’ Two Signal Model of lymphocyte activation, for the reasons of clarity that will become clear later.

I recapitulate here the reasons for proposing the one-lymphocyte/multiple-lymphocyte model for the inactivation/activation of lymphocytes, as I believe the underlying considerations are pertinent to contemporary issues. This model incorporates a mechanism of peripheral tolerance consistent with the ‘historical postulate’, proposed by Burnet and Fenner. According to the historical postulate, self-antigens are present before lymphocytes are generated. Consider the generation of those lymphocytes specific to a self-antigen, S. The first lymphocyte generated, specific to S, will be inactivated by S, as lymphocyte activation requires lymphocyte cooperation. Further lymphocytes specific for S will be inactivated as they are generated, one or a few at a time. Lymphocytes specific for a foreign antigen F will accumulate during ontogeny in the absence of F; when F later impinges upon the immune system, F can mediate the lymphocyte cooperation postulated to be required for lymphocyte activation.

These proposals, besides providing an explanation of peripheral self–nonself discrimination, accounted for some older, unexplained observations, collectively referred to as ‘carrier effects’ 17. Of particular importance were observations on ‘breaking’ the unresponsive state, made in the early 1960s 19,20.

It had been found that administration of large amounts of some foreign proteins to newborn animals, including rabbits, could make them unresponsive to a challenge of the antigen that in adult, naive animals readily produced antibodies. It was believed that these unresponsive states corresponded to the natural unresponsiveness of the immune system towards self-antigens, a belief that has stood the test of time. One such antigen was bovine serum albumin (BSA). Rabbits, exposed at birth to a large dose of BSA, were found to be unresponsive to BSA, as assessed by a challenge at 3 months of age, that generated a robust antibody response in naïve rabbits. The antigen human serum albumin (HSA) cross-reacts with BSA in rabbits to the tune of 15%, meaning that 15% of the antibody normally generated upon immunization of rabbits with HSA also binds to BSA. It was found that BSA-unresponsive rabbits, when challenged with HSA at 3 months of age, readily make detectable antibody, some of which bound to both HSA and BSA. Clearly, according to the Clonal Selection Theory, precursor cells of antibody-producing cells, with antibody receptors able to bind to both BSA and HSA, exist in 3-month-old, BSA-unresponsive rabbits. The critical question then was why can HSA, but not BSA, activate these anti-BSA/HSA antibody precursor cells? A natural explanation in terms of our model is that the BSA-specific lymphocytes are too scarce to cooperate, whereas there are many more lymphocytes specific to the ‘foreign’ antigen HSA. In this case, HSA can mediate lymphocyte cooperation and so induce the anti-BSA/HSA-specific precursor cells of the antibody-producing cells 18.

A number of similar observations can be similarly explained. These observations collectively have led to the recognition that peripheral tolerance to a peripheral self-antigen can sometimes be broken when a foreign antigen, that cross-reacts with the peripheral self-antigen, impinges upon the immune system. The occurrence of autoimmune rheumatic heart disease, following infection by group A streptococci, is recognized as being due to a cross-reaction between these bacteria and heart tissue 21. Thus, the Two Signal Model can account for how some autoimmunity arises.

I have deliberately given an account of the single-lymphocyte/multiple-lymphocyte model for the inactivation/activation of lymphocytes without considering the possibility, which we all know to be the case, that there are different lymphocyte subsets. I did this because I believe this exposition best exemplifies the essence and potential generality of the model.

Many observations, reported after the original Two Signal Model was proposed, can be interpreted as supporting its tenets. The activation of most B cells, identified as Lederberg's precursor of antibody-producing cells, requires antigen-specific CD4 T helper cells 22–24, and in their absence, antigen can inactivate the B cells 25,26. The efficient activation of at least some CD8 T cells, which gives rise to cytotoxic T lymphocytes (CTL), often critical in containing viral infections and cancer cells, requires help from antigen-specific CD4 T cells. In the absence of such help, antigen inactivates the CD8 T cells 27,28. Therefore, CD4 T helper cells appear to be the guardians over the fate of most B and CD8 T lymphocytes when they encounter antigen. The recognition of this generalization is in large measure why so much emphasis has been directed at trying to understand what determines whether antigen activates or inactivates CD4 T cells, and hence what controls the initiation of most immune responses. This is the major question I attempt to address in this forum.

(CA makes a very pertinent point. He states that primary CD8 T cell responses can occur in some systems in the absence of CD4 T cells, although the sustained generation of CD8 T cells, and the generation of memory CD8 T cells, appears to be CD4 T helper cell dependent. I acknowledge this. The question of the physiological significance of such responses and how they might occur mechanistically are questions I avoided in the original submission in order to be succinct. Some pertinent considerations concerning the activation of CD8 T cells seem very similar to considerations entertained below as I discuss the requirements to activate CD4 T cells, so I shall briefly respond to this issue later.)

## The activation and inactivation of CD4 T cells

To discuss the issues clearly, I feel it helpful to delineate my understanding of how certain models arose.

### The original Two Signal Model

Cohn and I predicted that the activation of T helper cells requires collaboration between helper T cells and that the interaction of antigen with a single precursor T helper cell results in its inactivation 18. We made this suggestion both because it seemed consistent with the admittedly very limited and indirect experimental evidence available 20, as I discuss later, and mainly because it provided a description of peripheral tolerance at the level of CD4 T cells consistent with the historical postulate. Two issues, arising from our proposals, could be regarded as problematic and were considered somewhat problematic by me 29. Firstly, were activated CD4 T cells required to activate naïve CD4 T cells, or could naïve CD4 T cells help their mutual activation? We opted for the first possibility 29 that activated CD4 T cells are much better at activating naïve CD4 T cells than are naïve CD4 T cells. This gave rise to the ontological question of how does the first activated CD4 T cell arise. I refer to this as the ‘priming problem’, for later reference and discussion. Secondly, the idea that the primary activation of CD4 T cells requires CD4 T cell cooperation somewhat stretches credibility, in that such cells are very scarce in an unprimed animal. How could such scarce cells find each other to interact? I refer to this as the ‘scarcity problem’, also for later reference.

### The Constitutive Model

Lafferty and Cunningham in the mid-1970s were the first to propose, on the basis of observation, that two signals are required to activate T cells. They found that the ability of MHC-bearing stimulator cells to induce MHC-disparate lymphocytes to mount a response to the MHC antigens of the stimulator cells did not correlate with the expression of MHC antigens on the stimulator cells. They found that strong expression of MHC antigens on stimulator cells is required but is insufficient; they postulated that good stimulators not only express the pertinent MHC antigens but have some additional property 30. Lafferty and colleagues referred to good stimulators as having an S^+^ phenotype 31. In time, in the late 1980s, partly through the observations of Quill and Schwartz, it became recognized that S^+^ cells were antigen-presenting cells (APCs) that *constitutively* express B7 molecules (CD80 and CD86), called costimulatory (CoS) molecules, recognized by the CD28 molecules on the surface of naïve T cells 32. Hence, my referring to this view as the Constitutive Model of CD4 T cell activation 6. The activation of a naïve CD4 T cell was envisaged to require signal 1 and a second costimulatory signal, following B7/CD28 interactions. Related observations led to the idea that the generation of signal 1 alone, for a sustained time of a day of two, could render the T cells anergic, unresponsive to restimulation 32. Activation of CD4 T cells required the generation of both signal 1 and the costimulatory signal, due to the CD28/B7 interaction.

Lafferty and Cunningham gave us, Bretscher and Cohn, credit for the idea of the need for two signals for lymphocyte activation 30. This civility caught on. Thus, the CD28/B7 costimulatory signal became identified in most people's mind as the molecular form of our signal 2, as far as the activation of CD4 T cells was concerned. I regarded this credit as somewhat unfortunate and was unsuccessful in my attempts to put matters right, by submitting letters outlining my view to appropriate journals. Cohn and I had envisaged that the delivery of ‘our’ signal 2, to the responding lymphocyte, only occurred following the recognition of antigen by a cooperating helper lymphocyte. What was known about the helper T cell-dependent activation of B cells and of CD8 T cells is in precise accord with our suggestions, as described previously and referred to as the original Two Signal Model. However, what one might call the Lafferty/Schwartz Two Signal Model of CD4 T cell activation was contrary to our suggestions. I personally expressed to Kevin Lafferty my concern. In a sense, I think the credit we mistakenly received was unfortunate; it may partially explain why so few took our original suggestion, with respect to the activation of CD4 T cells, seriously. It appeared to most that our predictions had been verified!

The Constitutive Model was historically important; it led to the recognition that CD4 T cell activation involved a Two Signal mechanism, but it did not readily explain what determines whether antigen activates or inactivates CD4 T cells. Embellishments of the model were needed to explain what factors/circumstances were critical.

### The pathogen-associated molecular pattern and Danger Models for the activation of CD4 T cells

Charles Janeway, in his opening Introduction to a Cold Spring Harbor Laboratory Meeting held in 1989, made some radical proposals 33. He pointed out how much of our knowledge, pertinent to the activation and inactivation of different lymphocyte types, was naturally accommodated by a Two Signal mechanism. Given the central role of CD4 T cells in determining whether antigen activates or inactivates other classes of lymphocyte, he focused on the requirements to inactivate/activate CD4 T cells. He noted that immunologists often employ foreign, vertebrate antigens when studying the regulation of the immune response, most often using microbe-containing adjuvants to generate immune responses. He referred to this practice as the ‘immunologists’ dirty little secret’ and suggested this secret, usually ignored at the conceptual level, was of fundamental significance. He proposed that the immune system does not discriminate self from nonself, but rather infectious entities from non-infectious entities. He postulated that pathogen-associated molecular patterns (PAMPs), of microbial/parasite origin, are required to initiate immune responses. These PAMPs were envisaged to interact with host pattern recognition receptors (PRR), resulting in the APC expressing appropriate amounts of the CoS molecules required to activate naïve CD4 T cells and so initiate an immune response 34.

A deficiency of Janeway's view was that it could not account for immune responses to vertebrate antigens, administered without microbial adjuvant, including transplants between different strains of mice. These antigens and tissues presumably do not express PAMPs. Matzinger broadened this theory by proposing that initiation of CD4 T cell activation could only occur under stressful circumstances, collectively referred to as ‘danger’ 35, including, I believe, those circumstances proposed by Janeway. These proposals have been very influential. I think it fair to say they represent the predominant, contemporary framework, with a caveat or two that I address later.

According to the proponents of the PAMP and Danger Models, most CD4 T cells, specific for peripheral self-antigens, are inactivated under steady-state conditions, when infections do not occur, or danger does not exist. Under such conditions, the number of CD4 T cells specific for peripheral self-antigens is kept lower than the numbers specific for a comparable foreign antigen. Under non-steady-state conditions, following an infection or danger, a few CD4 T cells specific for peripheral self-antigens might well be activated, but it is envisaged that they are likely inactivated once the infection has been cleared or the danger past, and so a return to the steady state occurs. It is significant in this context that activated CD4 T cells can be inactivated under steady-state conditions 36,37.

### The Two Step, Two Signal Model of CD4 T cell activation

I proposed this model in 1999 38. It was an attempt both to maintain the physiological advantages of the original Two Signal Model, to take account of more modern findings, and to face some of the issues/problems 29 I identified as arising from the original model. To be honest, these were the positive reasons, but I was also uncomfortable with the PAMP and Danger Models of CD4 T cell activation 6,38. I should like to start by summarizing my reservations, as only in the context of such reservations will my model not appear too extravagant.

Janeway's and Matzinger's proposals are to my mind radical. They suggest that the CD4 T cells of the immune system do not discriminate self from nonself, but rather situations where PAMPs or danger are present from situations where these triggers are absent. Consider two CD4 T cells emigrating from the thymus, one specific for a peripheral self-antigen, pS, the other specific for a foreign antigen, F, in the circumstances where both are present. According to the PAMP and Danger Models, whether pS and F activate their corresponding CD4 T cells depends only upon the circumstances *at this time*, not on the past history of the individual with regard to pS or F. The PAMP and Danger Models deny the existence of self–nonself discrimination at the level of peripheral CD4 T cells 33–35, as well as the pertinence of the historical postulate. These models discriminate between infectious/non-infectious entities and between dangerous/non-dangerous situations. It is unclear why the presence of PAMPs, or the existence of danger, would not sometimes allow both F and pS to activate their respective CD4 T cells, as neither PAMPs nor danger are antigen specific. To my mind, the facility with which these models thus account for the activation of CD4 T cells specific for peripheral self-antigens makes them somewhat implausible, in the absence of evidence to make these models overwhelmingly compelling. My unease led me to articulate a principle to clearly express the basis of my concerns. I suggest a reasonable principle, at the level of the system, would be that the mechanism by which a foreign antigen F activates its CD4 T cells should not, or at least should minimally, *interfere* with the mechanism by which pS inactivates its CD4 T cells. I have explored whether this ‘principle of non-interference’ can be incorporated into hypothetical mechanisms by which antigen activates and inactivates CD4 T cells, which are consistent with observation, as discussed below.

Another difficulty I have with the PAMP and Danger Models is that foreign, vertebrate antigens, presumably free of PAMPs, can be administered under non-dangerous conditions in a way that results in an immune response. This seems to me difficult to reconcile with this model 6,38.

Before elaborating on why I think my model is plausible, I wish to try to ‘take action’ to minimize a potential misunderstanding. There is overwhelming evidence that the presence of PAMPs and/or danger can affect the expression by APC of CoS molecules and do much else besides. I not only accept this evidence but welcome it. It makes eminent sense in evolutionary terms that, when infections occur or dangerous situations exist, these signals of alarm are exploited to regulate the immune response, controlling its rapidity and magnitude, for instance. However, whether such events occur is not the issue as I see it, as they clearly do and have a physiological role. The issue is whether PAMPs and danger are the *critical* elements in determining whether antigen activates or inactivates CD4 T cells, as proposed by Janeway and Matzinger 33–35. This seems implausible to me as a primary mechanism, due to the non-specific nature of PAMPs and danger. In addition, ‘giving’ pathogens the responsibility of turning on a critical immunological switch seems to be an abdication of responsibility, an abdication that could have unfortunate consequences.

**Figure 1:**
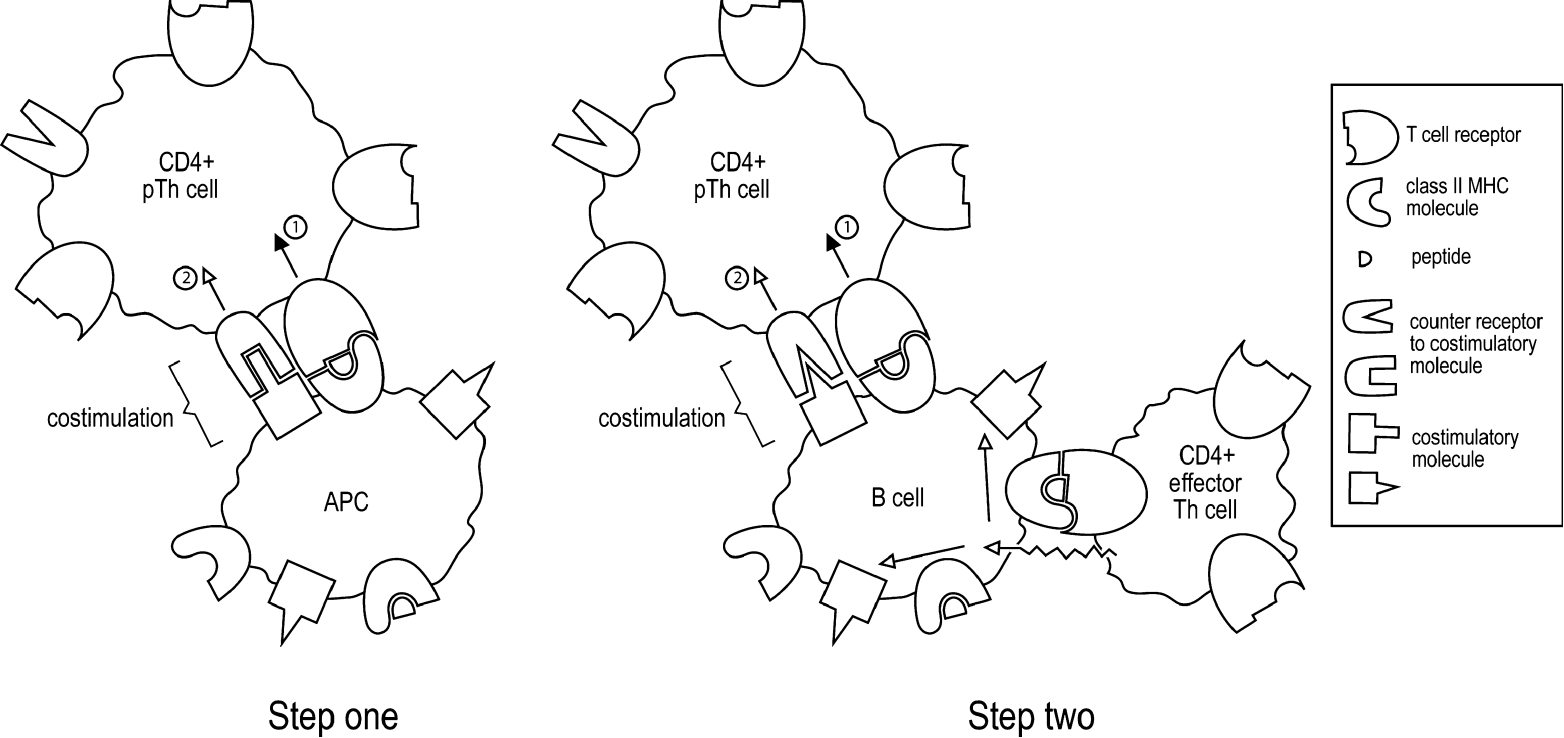
The Two Step, Two Signal Model of CD4 T cell activation. For a detailed explanation, see the text.

The Two Step, Two Signal Model I propose is illustrated in Fig.1. I now delineate its significant features.

1. In step one, a mature dendritic cell, or a macrophage, presents antigen to a naive CD4 T cell, a precursor T helper (pTh) cell, resulting in its multiplication. Its progeny can have some effector functions, such as the production of cytokines. Activation in step one requires the generation of two signals, as indicated. However, the costimulatory signal, or signal 2, is similar to the signal 2 of the Constitutive Model of T cell activation, as described previously, and is different in principle from the signal 2 of the original two-signal model, in that the initiation of its delivery does not follow from the recognition of the nominal antigen by a helper lymphocyte. To achieve the sustained activation of CD4 T cells, some of these CD4 T cells, arising from step one, must complete step two. A question naturally arises from an examination of Fig.1. What is the origin of the CD4+ effector Th cell depicted in step two? This is addressed below, see under section ‘The priming problem’.
2. The step one activated CD4 T cells are fully activated if, in time, they complete step two, during which the CD4 T cell again receives two signals. The ‘costimulatory signal’ of step two must be somehow different from the costimulatory signal of step one, if step two is envisaged, as I envisage, to be required to result in the full and sustained activation of CD4 T cells. One possibility is that the costimulatory molecule and its counter-receptor are different in step one and step two, a possibility depicted in Fig.1.
3. The APC in step two is an antigen-specific B cell that presents peptides that are derived from the nominal antigen for which the B cell is specific. This B cell will only express the requisite CoS molecules if activated by an activated antigen-specific CD4 T cell. Thus, step two requires the collaboration of three lymphocytes specific for the same nominal antigen. If this step is not completed, the activation of step one CD4 T cells is not sustained, and they are silenced (die or become anergic). The envisaged model reincorporates, admittedly in a more elaborate form, the one-lymphocyte/multiple-lymphocyte idea for the inactivation/activation of lymphocytes and thus accounts for peripheral tolerance of CD4 T cells.

The proposal that the APC in step two is an antigen-specific B cell was made for three reasons. Both macrophages and DC can take up multiple antigens and present peptides derived from the processing of these diverse antigens. In contrast, B cells preferentially take up the antigen for which they are specific. The requirement for a specific B cell, as the APC in step two, minimizes the ability of CD4 T cells, specific for a foreign antigen F, to help the activation of CD4 T cells specific for pS, thereby subverting the inactivation of CD4 T cells specific for pS. Thus, the proposal that antigen-specific B cells mediate CD4 T cell cooperation allows a realization of the ‘principle of non-interference’. There is a second consequence of the APC in step two being a B cell. B cells specific for pS are usually deleted, and so step two usually cannot take place for pS-specific CD4 T cells. In this case, pS-specific CD4 T cells will be inactivated. Thirdly, the frequency of antigen-specific B cells and CD4 T cells in an *unprimed animal* is comparable, and much less frequent than professional APC such as DC and macrophages. It would seem, if the frequency of the APC, present in a naïve animal and presenting the pertinent antigen, was very much greater than the frequency of the interacting CD4 T cells, that the antigen-presenting cells would be in such excess that these APCs would not be able to efficiently mediate CD4 T cell interactions. We discuss below how the postulated involvement of B cells might be important in understanding the efficacy of a treatment of autoimmunity.

(AC points out that the envisaged requirement for B cells to activate CD4 T cells involved in cell-mediated immunity means there cannot be a cell-mediated response to antigens not recognized by B cells. He points out that cell-mediated responses are often generated against intracellular pathogens, and antibody responses to extracellular antigens. This leads to the question of how do Th cells specific for intracellular antigens get activated?

In view of the envisaged role of B cells, it must mean, if this idea is correct, that significant extracellular antigen arises from intracellular infections. We actually know this is most probably the case from another consideration; the presence of extracellular antigen is required for the generation of Th1 cells that recognize exogenous antigen presented by APC).

### The scarcity problem

This problem has become even more critical in this model, from one perspective, than it was in the original Two Signal Model. The scarcity problem was recognized when it was envisaged that two scarce CD4 T cells had to find each other to interact to initiate an immune response. In the case of the model under consideration, it is proposed that three scarce cells must interact, two CD4 T cells that collaborate via an intermediate, antigen-specific B cell.

Two considerations mitigate my concern. The existence of step one allows for the clonal expansion of the naïve CD4 T cells before the ‘two’ CD4 T cells need to interact. The only limitation on the expansion of a single CD4 T cell is that it must not be so extensive that its progeny, by themselves, constitute a ‘cooperative group’, able to complete step two. Secondly, since the model was made, imaging studies have provided a vivid picture of how the model could be physically realized. Naïve CD4 T cells enter a lymph node via the high endothelial venules, near which they can first interact with antigen presented by a professional APC, a macrophage or DC, resulting in their multiplication. After 2 or 3 days, they migrate in a directed fashion to the B cell/T cell boundary, where they appear to interact with antigen presented by B cells 39,40. I consider these observations as in striking accord with the model.

### The priming problem

The priming problem is resolved in this model by the postulate that the interaction between CD4 T cells does not occur in a first critical step, but rather later, in step two, acting as a ‘checkpoint’, after step one.

## Comments in response to those of the reviewers

I try to outline here various substantive comments made by CA and AC, and my response to these comments. I have tried to collect their concerns/questions into groups that I feel call for related responses. These comments/responses by CA and AC, and by myself, already constitute part of a significant discussion.

### A request made by both CA and AC was for examples of the activation of CD4 T cells without PAMPs or danger

I have in the past given the example of sheep RBC (SRBC), delivered without adjuvant and via a sharp needle, to mice, in ‘Seminars in Immunology’ 6. This forum involved several rounds of discussion in which invited people expressed their written thoughts on a topic, with all participants seeing each other's contributions before the next round. Colin Anderson and Polly Matzinger proposed in the last round, in response to one of my questions, that the response to SRBC was really in some sense a secondary response 41. They quoted a paper of mine 42 in which I noted that there are quite high numbers of SRBC-specific antibody-producing cells in mice not immunized by immunologists. They suggested that the antibody/SRBC complexes could bind, via Fc receptors, to DC, thereby activating the DC, this presumably constituting one kind of danger signal. This proposal is one to which I shall return, see 2, under section ‘The importance of Non-Interference in the context of the different models’. However, my paper also showed that rat red blood cells (RRBC) are also immunogenic. RRBC can induce both the formation of antibody and the expression of DTH, but I could not detect any RRBC-specific antibody-producing cells in the spleen of unimmunizing mice employing very powerful, single-cell assays. Thus, the explanation for the response induced by SRBC probably does not apply to the response to RRBC, and thus perhaps also not to the response to SRBC.

A second example seems even more interesting. It appears to convincingly demonstrate in a general way that neither PAMPs nor danger signals are required to activate CD4 T cells.

We defined the peptide specificity of virtually all the CD4 T cells generated in the spleen upon immunizing BALB/c mice with the antigen, hen egg lysozyme (HEL). About half the CD4 T cells are specific to the ‘major peptide’ of HEL, HEL_105–120_ 43. It was very difficult to generate responses to HEL without employing microbial adjuvants, but we managed to do so. We envisaged that this difficulty likely reflected the ‘scarcity’ of CD4 T cells specific for HEL, and designed experiments based upon this surmise. We administered the major peptide of HEL to BALB/c mice in a manner known to ablate their CD4 T cells, that is, by giving a series of high doses of the peptide iv. We subsequently gave these mice, and control mice, a standard challenge of HEL 44. Not unexpectedly, the experimental mice did not produce CD4 T cells specific for the major peptide on this challenge. However, their production of CD4 T cells specific for the minor HEL peptides was also dramatically reduced! This seems to show that CD4 T cells specific for the major peptide facilitate the activation of the CD4 T cells specific for the minor peptides. Control experiments make it highly unlikely that the lack of response in the experimental mice was due to inhibitory T cells 44.

I think these experiments can also be employed to argue against the PAMP and Danger Models 44. The control mice, on HEL challenge, produced CD4 T cells specific for the major and for the minor HEL peptides. This challenge must therefore contain PAMPs or generate danger, according to the hypothesis entertained. The same LPS-decontaminated HEL challenge was employed to immunize the experimental mice. The lack of production of CD4 T cells specific for minor HEL peptides in the experimental mice seems to me difficult to reconcile with the PAMP and Danger Models. This experiment also nicely illustrates the pertinence of the historical postulate in the context of the one-lymphocyte/multiple-lymphocyte model for lymphocyte inactivation/activation. Prior ablation of CD4 T cells specific for the major peptide undermines the production of CD4 T cells specific for another peptide on a challenge with antigen whose processing results in the presentation of both peptides.

### Clarification on what I think are the requirements for the expression of costimulatory molecules by the APC involved in step one

#### Request made by both CA and AC

My view is that different types of APC exist to serve different physiological functions. Thus, the well-recognized characteristics of mature DC, namely being minimally phagocytic, expressing peptides derived from exogenous antigens brought into the dendritic cell by various means when the dendritic cell is in an immature state, their greater expression of CoS than when immature, and the DC's migration to the draining lymph node on maturation, seem most appropriate characteristics for the mature DC's role of providing information, to the draining lymph node, of the universe of antigens present at the site of the DC's maturation. It is clear that PAMPs have a role in at least accelerating the process of maturation.

Macrophages have somewhat different properties, ideally suited to dealing with systemic infections. Thus, macrophages are both competent as APC and have the ability to phagocytose antigen. They thus provide an ongoing means of reporting to CD4 T cells on the universe of antigens in the systemic environment. I shall later address some possible implications of these differences between DC and macrophages.

I gather that DC may be more in people's thoughts than macrophages when they think about the ‘steady state’, which may be significant, as I discuss below, under the section ‘The importance of Non-Interference in the context of the different models’.

### The importance of Non-Interference in the context of the different models

CA suggested that I am overstating the advantages of the model I favour in the context of ‘The Principle of Non-Interference’ and that this is a most critical point. I agree with the criticality of the issue and so will try to address it at some length.

I have four points I would like to make in this context.

(1) An important issue is whether PAMPs or danger signal are just needed to initiate the activation of CD4 T cells, or whether their sustained activity is needed to maintain CD4 T cell activation. It seems clear to me from the literature 41, and from his comments as a referee, that CA favours the sustained option. I will therefore just consider this scenario.

There are ongoing CD4 T cell responses to most foreign antigens in the spleen and most likely in other secondary lymphoid organs. Single-cell assays for detecting *ex vivo* antigen-specific, cytokine-producing CD4 T cells 45 support the presence of such cells specific for most foreign antigens in the spleen of mice not immunized by immunologists (P. Bretscher, unpublished observations). The presence of such cells is somewhat analogous to the existence in the spleen of low numbers of antibody-producing cells specific for most antigens, as again detected by single-cell assays 42, presumably reflecting ongoing immune responses to a diversity of antigens. There must therefore be widespread PAMPs or danger signals to sustain the generation of these activated CD4 T cells, according to PAMP and Danger Models. Moreover, peripheral self-antigens are usually replenished, and so continually present, and so may be taken up by immature DC and by macrophages that are phagocytic when mature, leading to the generation, and sustained generation, of autoimmunity.

(2) I would like to return to the situation envisaged by Colin Anderson and Polly Matzinger to explain the immunogenicity in mice of SRBC, namely that this response is in a sense a secondary response, facilitated by the activation of APC by antigen/antibody complexes.

Consider a peripheral self-antigen, continuously present at a low level, against which, following an acute infection, some antibody has been produced, and some CD4 T cells have been generated. According to my understanding of the situation Colin and Polly envisage, this circumstance would most probably give rise to sustained activation of CD4 T cells specific for this peripheral self-antigen. Thus, the generation of autoreactive responses, which included the generation of activated CD4 T cells and the production of antibody, following an acute infection, would often be sustained.

Moreover, the macrophages and immature DC may take up diverse antigens. In this case, antibody/antigen complexes could activate the APC that could present peptides derived from diverse antigens, including peripheral self-antigens.

(3) It is my feeling that, as immune responses are pretty ubiquitous, danger must be fairly readily generated and, in this sense, circumstances would often arise where CD4 T cells specific for peripheral self-antigens are induced, in terms of the Danger Model. The conditions under which autoimmune CD4 T cells are likely to be activated, in terms of the Two Step, Two Signal Model, are, I believe, much more restricted and also more defined. I would like to give three examples of rather particular circumstances where ‘autoreactive CD4 T cells’ can be generated. These particular and therefore limited circumstances can, I believe, be readily understood in terms of the Two Step, Two Signal Model.

#### Example 1

The studies of Weigle, carried out in the early 1960s 19,20, were pivotal for me in trying to understand the basis of peripheral tolerance in the late 1960s. They are well worth revisiting from a modern perspective, despite the fact that much detailed analysis was not, and could not, be carried out at that time. I might add that these studies still fascinate me from the perspective of what inspired them. Weigle did not interpret his findings in a coherent manner, but it seems to me, from the experiments he did, he must have had an intuitive insight for what is important. I have always felt he was not given sufficient credit for his contributions.

I have already delineated the type of system Weigle employed. Rabbits given very high doses of BSA neonatally were unresponsive to a challenge of BSA, at 3 or 6 months of age, but did respond to a challenge with HSA at 3 months. These two antigens cross-react about 15% as assessed with rabbit antisera. Some of the antibodies produced in BSA-unresponsive rabbits, upon immunization with HSA, bound to BSA. All recognize that this means, in modern terms, that there are, in 3 month old, BSA-unresponsive rabbits, B cells with receptors that can bind to both BSA and HSA. All agree that the ability of HSA, in contrast to BSA, to induce these B cells is because HSA can efficiently induce HSA-specific CD4 T cells.

Weigle also undertook further experiments, in which he challenged BSA-unresponsive rabbits with other foreign serum albumins in the same manner. Of particular interest was the challenge with sheep serum albumin (SSA). Weigle reported that SSA and BSA cross-react, as assessed with rabbit antisera, to the tune of 75%. Challenge of BSA-unresponsive rabbits with SSA at 3 months of age, did not result in the production of antibody: none that bound to both BSA and SSA and none that bound only to SSA. Consider how we might explain this in modern terms. As BSA and SSA are not identical, there must be some SSA-specific CD4 T cells in BSA-unresponsive rabbits. These SSA-specific CD4 T cells are clearly not efficiently activated by a challenge of SSA, whereas the HSA-specific CD4 T cells in BSA-unresponsive rabbits are efficiently activated by HSA. It seems likely this is because there are more HSA-specific lymphocytes than there are SSA-specific lymphocytes in BSA-specific unresponsive rabbits. According to the Danger Model, danger is most likely similarly present when the BSA-unresponsive rabbits are similarly challenged with HSA or with SSA. The better ability of HSA than SSA to activate antigen-specific lymphocytes would presumably reflect their greater frequency, perhaps fourfold, given the degree of cross-reactivity of HSA (15%) and SSA (75%) with BSA. In contrast, there is expected to be a minimum number of CD4 T lymphocytes required to initiate sustained CD4 T cell activation by antigen on the Two Step, Two Signal Model, and so the very different outcomes on challenge with HSA and SSA are more readily understood.

Further experiments by Weigle reinforce these inferences. They support the idea that these experiments, through their assessment of the production of antibody, really reflect requirements for the induction of CD4 T cells. Weigle showed that, by immunizing BSA-unresponsive rabbits repeatedly with HSA, he could render them able to respond to a subsequent challenge with BSA to produce anti-BSA antibody 20. If this production of BSA-specific antibody is dependent on BSA-specific CD4 T helper cells, as seems most likely, it must mean that a few BSA-specific CD4 T cells exist in 3-month-old BSA-unresponsive rabbits. Some of these BSA-specific CD4 T cells are better activated by HSA than by BSA. This is naturally explained if CD4 T cell helper activation requires lymphocyte cooperation!

#### Example 2

It is well recognized that infection by group A streptococci can result in rheumatic heart disease, due to a cross-reaction between this bacterium and heart tissue, associated with the production of antibodies to heart tissue. All understand that the production of anti-heart tissue antibody is due to the activation of cross-reacting B cells that are helped by streptococci-specific CD4 T cells 21. It has been demonstrated that infection also results in the activation of CD4 T cells that react with both streptococcal antigens and heart tissue 46. Why are these cross-reactive CD4 T cells better induced by streptococci than by the heart tissue itself? The Two Step, Two Signal Model predicts that immune responses for foreign antigens, which cross-react with peripheral self-antigens, can result in the activation of CD4 T cells specific for these peripheral self-antigens, but not CD4 T cells specific for other peripheral self-antigens. It seems to me the Danger Model is much less specific; if sufficient danger is generated by a streptococcal A throat infection, it might be anticipated that autoimmunity to diverse peripheral self-antigens would be generated. Thus, it is more readily understandable on the Two Step, Two Signal Model that the first autoimmune CD4 T cells generated react to both the peripheral self-antigen and the inciting antigen.

#### Example 3

Charles Janeway and colleagues examined the circumstances under which mouse cytochrome *c*-specific CD4 T cells can be generated in mice. It is interesting that Janeway first championed the PAMP Model of CD4 T cell activation in 1989 33. The experiments I describe here were published in 1991 47. Janeway's report gave me encouragement to propose the Two Step, Two Signal Model, in 1999, partly as a result of my unease with Janeway's own proposals!

These studies show that immunization of mice with human cytochrome *c* can result in antibodies that recognize both human and mouse cytochrome *c*. Janeway and colleagues showed in addition that B cells, from mice primed with human cytochrome *c*, would allow, when transferred to recipient mice, these mice to generate mouse cytochrome *c*-specific CD4 T cells on immunization with mouse cytochrome *c* given in complete Freund's adjuvant (CFA). Thus, some mouse cytochrome *c*-specific CD4 T cells must exist in these mice. Yet, immunization with mouse cytochrome *c* in CFA was insufficient to activate these mouse cytochrome *c*-specific CD4 T cells. This observation seems paradoxical in terms of the PAMP and Danger Models. The requirement for activated mouse cytochrome *c*-specific B cells is, however, consistent with a central role of activated B cells in the activation of CD4 T cells, as envisaged in the Two Step, Two Signal Model.

### Activation of CD8 T cells to produce cytotoxic T lymphocyte responses without CD4 T cells

This section is written in response to comments made by CA and already referred to.

It seems that in different systems the activation of different facets of the CD8 CTL response can be more or less CD4 T cell dependent. I feel three points are pertinent.

The central effector role of CTL in fighting various infections, particularly viral infections, means there must be an evolutionary premium on both generating rapid CTL responses upon infection and yet having a means of avoiding anti-self CTL responses getting out of hand. These dialectical needs are rather similar to the situation we have faced when considering the activation of CD4 T cells. Secondly, there likely is an initial phase of the activation of a CD8 lymphocyte that is lymphocyte cooperation independent, resulting in very rapid responses 48, with a later checkpoint step that is lymphocyte cooperation dependent, curtailing responses against antigens for which there are initially only a few lymphocytes. Thirdly, some apparent discrepancies might be accounted for by the following considerations. As some activated CD8 T cells do produce significant IL-2 and express such activation-dependent surface antigens such as CD40L, they have some of the characteristics of helper lymphocytes, most prominently displayed by activated CD4 T cells. Indeed, there is evidence that CD8 T cells can help each other to expand through their production of IL-2 49. In those cases where there are sufficient CD8 T cells to satisfy any checkpoint involving lymphocyte cooperation, we would have CD8 T cell responses that are CD4 T cell independent. In other cases, there might be insufficient CD8 T cells to mutually sustain their activation, and so the CD8 T cell response would be CD4 T cell dependent. In addition, as early phases of activation may be less lymphocyte cooperation dependent than later stages, early stages may be CD4 T cell independent and later stages CD4 T cell dependent.

### Negative regulation of CD4 T cell activation

I would like to briefly attempt to respond to various further comments expressed by CA in the context of the proposals I made. CA was concerned that I misrepresented the predominant, contemporary view as to how CD4 T cells are inactivated. CA made two points. Firstly, he pointed out that I have avoided addressing the role of T_reg_ cells, believed by many to be important in suppressing the activation of self-reactive lymphocytes. I would like to defer the rather limited comments I can make on this topic to the second forum, on immune class regulation 1. Secondly, CA felt I had completely avoided the role of coinhibitory signals involved in CD4 T cell inactivation.

It is well appreciated that activated T cells express CTLA-4 on their surface and that this molecule has a greater affinity for B7 molecules than does CD28. Thus, CTLA-4 acts at least as a feedback regulator on CD4 T cell activation. Given the spiral inherent in the proposal that helper lymphocytes help the activation of helper lymphocytes, we would have uncontrolled helper lymphocyte expansion, in the sustained presence of antigen, in the absence of feedback control. Mice that are engineered not to express CTLA-4 have a lymphoproliferative disorder and die at a few weeks of age. These findings implicate CTLA-4 as central to a major feedback mechanism on T cell activation 50. There are reports that the inactivation of CD4 cells can be prevented by blocking CTLA-4 51, or a related molecule PD-1 52, expressed by activated CD4 T cells and other cells. I have a difficulty in developing a coherent and detailed picture of the roles of CTLA-4 and PD-1 and their ligands in the inactivation of activated CD4 T cells. Perhaps, CD4 T cell collaboration results in signals to the target CD4 T cell that downregulates CTLA-4 and/or PD-1 expression by this target CD4 T cell, thus allowing the target cell to survive and thrive.

A response to a last comment made by AC. I had stated that the Two Step, Two Signal Model had the requirement that ‘the expansion of a single CD4 T cell does not give rise to a cooperative group’ of CD4 T cells that can complete step two. AC found this problematic. I was unsure why. There presumably can be internally controlled constraints on the number of cell divisions a naïve CD4 T cell will undergo, after successfully proceeding through step one. So long as the number generated is below the number of CD4 T cells required to have a significant likelihood of completing step two, we are in a position where the principle of one lymphocyte/multiple lymphocytes for lymphocyte inactivation/activation can be realized.

The constraints on the T cell number likely include both CTLA-4 and PD-1. Disruption of the interactions of inhibitory receptors, present on activated CD4 T cells, with their ligands may allow the progeny, resulting from a single CD4 T cell completing step one, to form a cooperating group of CD4 T cells that can collectively complete step two. In this case, the envisaged mechanism of peripheral tolerance for CD4 T cells would be subverted.

## Supporting/non-supporting evidence

I feel it inappropriate to critically consider here the evidence for or against the Two Step, Two Signal Model. I briefly indicate some key observations and pertinent references.

Several studies show that CD4 T cell activation is facilitated by CD4 T cell collaboration 44,53–57. This collaboration, between CD4 T cells, is most likely mediated by B cells acting as APC 56. It is found in both animals and humans that the CD4 T cell repertoire expands during the course of responses, and this can be particularly evident in autoimmune responses, where the antigen/peptide specificity of the first CD4 T cells detected is very restricted. Thus, the diversity of peptides recognized by autoimmune CD4 T cells expands with time 58,59. This phenomenon, referred to as ‘epitope spreading’, is consistent with the idea that CD4 T cell activation is facilitated by CD4 T cell cooperation. The ablation of the CD4 T cells, first appearing in an autoimmune response, prevents the whole cascade of diverse CD4 T cells from developing 58. This abrogation of CD4 T cell activation is anticipated on the model being discussed, but not so specifically on the PAMP and Danger Model for CD4 T cell activation.

One other class of observation is of particular interest for its potential relationship to the model I propose and for its clinical significance. It appears that the depletion of B cells, by administering a B cell-depleting antibody, can be beneficially employed to treat autoimmune disease 60–62. This includes diseases in which the damaging response is believed to be of a predominantly cell-mediated, Th1 nature. Studies in mice have led to the conclusion that the critical role of the B cell in such situations is not the production of antibody 63. This conclusion is consistent with the idea of a critical role of B cells as APC in activating CD4 T cells and in the process of epitope spreading. These studies make sense in terms of the model outlined, but are otherwise somewhat surprising.

Another set of observations is consistent with the Two Step, Two Signal Model, as well as the PAMP and Danger Models, and is outlined here for both its intrinsic interest and its possible utility in immunological intervention. These observations are on the conditions that allow both naïve and activated CD4 T cells to be inactivated.

Peptides bound to the grooves of class II MHC molecules have a half-life of the order of a day. It is possible to expose APC to class II MHC-binding peptides for some hours, resulting in the APC being ‘decorated from the outside’, when these peptides bind to ‘vacated grooves’. It is found that the systemic administration of large amounts of peptide to mice, or the systemic expression of antigen on DC, results in the inactivation of both the corresponding naïve and activated CD4 T cells 36,37,64. This result is expected on the PAMP and Danger Model, if the large majority of APCs are not activated by PAMPs or danger. It is also expected on the Two Step, Two Signal Model, as the decorated APC, including B cells, will, under most circumstances, be present in much greater numbers than the number of specific CD4 T cells, and so not be able to efficiently mediate CD4 T cell interactions.

Lastly, some studies appear inconsistent with the ideas underlying the Two Step, Two Signal Model. One such study by Anderson, Matzinger and colleagues was partially directed at critically testing whether the historical postulate applies to the peripheral tolerance of T cells 65. These investigators grafted H-Y bearing male skin onto immunoincompetent female mice, allowed the grafts to heal and then reconstituted the host with foetal liver, stem cells. The male skin was present before any lymphocytes developed, and so tolerance should have been established according to the historical postulate. However, the grafts were rejected. This is certainly contrary to predictions I would have made.

Further observations, made in related systems 66, might allow someone, who is partial to the historical postulate, such as myself, to argue that the further observations can be employed to ‘rescue’ the historical postulate. Colin Anderson and colleagues found that grafts with a single minor histoincompatible antigen, grafted to an internal body site, induced tolerance, whereas grafts with multiple minors did not. It seems to me that this shows a quantitative limit on the one-lymphocyte/multiple-lymphocyte model for the inactivation/activation of lymphocytes. If too many lymphocytes are generated within a short period of time, particularly in a lymphopenic environment, T cell inactivation does not occur.

I would like to make a philosophical point at this juncture. No description one can invent will incorporate all the subtleties and niceties of mother nature, so one should always anticipate that paradoxes will arise within a framework one develops; no framework is complete. This is not a statement against rigour but rather an acknowledgement of reality. Although some scientists might be critical if your view is not consistent with all the ‘facts’, I personally feel I am onto something if a physiologically appealing and coherent model is consistent with a large majority of the observations at hand. I anticipate that further understanding and embellishments of any model will be required. I therefore always consider models to be *minimal* models and seek for contradictions as a source of inspiration for further insight. I think the observations, just described and reported by Colin Anderson and his colleagues, demonstrate a limit under which the one-lymphocyte/multiple-lymphocyte model for the inactivation/activation of lymphocytes operates, particularly under lymphopenic conditions.
